# Multimodal Assessment of Bottlenose Dolphin Auditory Nuclei Using 7-Tesla MRI, Immunohistochemistry and Stereology

**DOI:** 10.3390/vetsci9120692

**Published:** 2022-12-13

**Authors:** Ksenia Orekhova, Enna Selmanovic, Rita De Gasperi, Miguel A. Gama Sosa, Bridget Wicinski, Brigid Maloney, Alan Seifert, Akbar Alipour, Priti Balchandani, Tommaso Gerussi, Jean-Marie Graïc, Cinzia Centelleghe, Giovanni Di Guardo, Sandro Mazzariol, Patrick R. Hof

**Affiliations:** 1 Department of Comparative Biomedicine and Food Science, University of Padova AGRIPOLIS, Viale dell’Università 16, 35020 Legnaro, Italy; 2 Nash Family Department of Neuroscience, Friedman Brain Institute, Icahn School of Medicine at Mount Sinai, New York, NY 10029, USA; 3 Department of Psychiatry, Icahn School of Medicine at Mount Sinai, New York, NY 10029, USA; 4 Friedman Brain Institute, Icahn School of Medicine at Mount Sinai, New York, NY 10029, USA; 5 Research and Development Service, James J. Peters Department of Veterans Affairs Medical Center, Bronx, New York, NY 10468, USA; 6 General Medical Research Service, James J. Peters Department of Veterans Affairs Medical Center, Bronx, New York, NY 10468, USA; 7 Laboratory of Neurogenetics of Vocal Learning, Rockefeller University, New York, NY 10065, USA; 8 Department of Radiology, BioMedical Engineering and Imaging Institute (BMEII), Graduate School of Biomedical Sciences, Icahn School of Medicine at Mount Sinai, New York, NY 10029, USA; 9 Faculty of Veterinary Medicine, University of Teramo, 64100 Teramo, Italy

**Keywords:** cetaceans, animals, stereology, MRI, immunohistochemistry, human, neurodegeneration, chronic traumatic encephalopathy, hypoxia, amyloid β, fibronectin, microglia, astrocytes

## Abstract

**Simple Summary:**

Monitoring cetacean health is important considering the high strain from both natural and human-related threats. Most of these, such as *Cetacean morbillivirus* and man-made pollution in form of toxins and noise, affect nervous tissues and brain function. Neuropathological research in cetaceans has been qualitative or semiquantitative. Here, we use stereology to quantify protein expression in neurons, axons, and glial cells in the auditory nuclei of a bottlenose dolphin and correlate the values to their pre-processed volumes from MRI scans. This multimodal approach is aims to avoid artifacts that may arise using either methodology. Similarities in protein expression between a healthy dolphin and a human with brain trauma implies that dolphins might have a baseline neurochemical buffer against low oxygen (hypoxia). This study will help expand our quantitative understanding of health and disease in cetacean brains.

**Abstract:**

The importance of assessing neurochemical processes in the cetacean brain as a tool for monitoring their cognitive health and to indirectly model human neurodegenerative conditions is increasingly evident, although available data are largely semiquantitative. High-resolution MRI for post-mortem brains and stereology allow for quantitative assessments of the cetacean brain. In this study, we scanned two brains of bottlenose dolphins in a 7-Tesla (7T) MR scanner and assessed the connectivity of the inferior colliculi and ventral cochlear nuclei using diffusion tensor imaging (DTI). Serial thick sections were investigated stereologically in one of the dolphins to generate rigorous quantitative estimates of identifiable cell types according to their morphology and expression of molecular markers, yielding reliable cell counts with most coefficients of error <10%. Fibronectin immunoreactivity in the dolphin resembled the pattern in a human chronic traumatic encephalopathy brain, suggesting that neurochemical compensation for insults such as hypoxia may constitute a noxious response in humans, while being physiological in dolphins. These data contribute to a growing body of knowledge on the morphological and neurochemical properties of the dolphin brain and highlight a stereological and neuroimaging workflow that may enable quantitative and translational assessment of pathological processes in the dolphin brain in the future.

## 1. Introduction

The dolphin brain is subject to a lot of scientific interest for several reasons, although many aspects of its neurochemical functions and connectivity remain unexplored. It is an example of convergent evolution between artiodactyls and humans in terms of their large brain size and extensive gyrification [1], the longevity of their individuals, the development of age-related degenerative conditions, and similar pathological reactions following exposure to infections, toxins, and certain intensities and frequencies of sound [2,3,4,5,6].

Under baseline conditions, the dolphin brain is exposed to variable and prolonged periods of limited oxygen during diving. It could therefore display characteristics of human brains that have been exposed to chronic trauma and neurochemical changes consistent with hypoxia, as in the case of patients with chronic traumatic encephalopathy (CTE) [7]. 

Several lines of research hint at morphological and molecular adaptations of the cetacean nervous tissues to buffer the strain of deep diving in a cool aquatic environment under high pressure, including the evolution of cervical rete mirabilia that ensure steady blood flow [8], higher levels of myelination [9], and antioxidant proteins [10] in deeper- versus shallow-diving cetacean species. Comparatively high levels of uncoupling proteins are hypothesized to aid in non-shivering thermogenesis to preserve brain function under cold ambient temperatures [11], although this hypothesis has been disputed [12].

Our recent work focused on validating proteins as indices of the health status of bottlenose dolphin (*Tursiops truncatus*) brains, particularly in the dubious cases of potential anthropogenic acoustic trauma [5]. However, only a semiquantitative approach was available at the time. In the current study, we focused on stereological estimates of validated neuronal and glial proteins in the inferior colliculus (IC) and ventral cochlear nucleus (VCN) of a bottlenose dolphin and outline a workflow that could be used in further studies of these and other brain areas. A similar approach was taken by Nieder and colleagues (2022) [13] when examining neuronal numbers in the lateral superior olivary nucleus of short-beaked common dolphins (*Delphinus delphis*).

We refer to 7T-MRI and DTI data to assess the volumes and connectivity of the auditory nuclei of two bottlenose dolphins, one of which was used for quantitative immunohistochemical assessment. This multimodal approach may help to circumvent artifacts due to the opportunistic nature of cetacean brain recovery and sampling, particularly with respect to effects of processing-induced tissue shrinkage.

Neuronal proteins, such as amyloid-β (Aβ) protein, associated with human neurodegenerative diseases, such as Alzheimer’s disease (AD), have already been used in previous cetacean brain studies. They are conversely linked to tolerance to hypoxia [5,14], and are quantified herein alongside TAR DNA-binding protein 43 (TDP-43)—another protein aggregating in diseases such as amyotrophic lateral sclerosis, Parkinson’s disease, and frontotemporal dementia [15]—and the extracellular matrix protein fibronectin. TDP-43 was previously observed in the brains of seven common dolphins with known methylmercury and cyanotoxin intoxication [4]. Fibronectin was primarily intended to visualize vascular and capillary networks, as it is an important constituent of the extracellular matrix of the vascular basal membrane [16,17], and capillary length and density are known to change under metabolic stress conditions such as hypoxia [18,19]. Fibronectin also plays a role in the extracellular matrix surrounding neurons and facilitates repair following injury [20].

SMI-312, an antibody targeting phosphorylated medium and heavy neurofilament fibers, was used to quantify axon length and density, while Iba-1 and GFAP were used to quantify microglial and astrocytic cells, respectively. Furthermore, qualitative trends between fibronectin expression in human CTE are observed in comparison to dolphin and healthy human tissues.

The aim of this study was to provide the first quantitative estimates of neuronal, axonal, and glial cell populations within the dolphin IC and VCN, outlining a protocol for stereological probe design and limitations that could serve as a platform for standardized quantitative comparisons between different individual animals and species.

## 2. Materials and Methods

### 2.1. Specimens

The brains from bottlenose dolphins used in this study were obtained from the Peter J. Morgane Collection on the Cetacean Brain. Marine 0116 was a 15-year-old female from an aquarium whose death in 1995 was associated with liver disease and pancreatitis. Its brain consisted of both hemispheres cut mid-sagittally and was stored in 4% paraformaldehyde solution at 4 °C. The left hemisphere was available from another animal, Marine 0142, and was stored in 10% neutral-buffered formalin at 4 °C. No further information was available on Marine 0142′s history, but the size of the brain was somewhat larger than that of Marine 0116, implying that it was extracted from an adult dolphin. A separate bottlenose dolphin brain (Dolphin 2) was studied using archived coronal Nissl-stained and celloidin-embedded sections (for more information, refer to Hof and Van der Gucht, 2006 [21]). Neuronal morphology was assessed using Nissl-stained sections in all three dolphins, and in Marine 0116 and 0142 the MRI scans were assessed, yielding no obvious pathology. No animals were euthanized for this study.

Archived tissues from three human brains were used for comparisons and as controls: one healthy male individual (74 years, 5 h post-mortem interval, Clinical Dementia Score 0 (=control), Braak NFT stage I), one with AD and severe cognitive impairment (male, 85 years old, 11 h post-mortem interval, Clinical Dementia Rating 3, Mini-Mental State Exam 11, Thal amyloid stage 4, Braak tangle stage V), and one with advanced CTE and moderate hypoxic-ischemic encephalopathy (male, 69 years old, 9 h post-mortem interval, moderate cerebrovascular disease and athero-arteriolosclerosis; severe post-mortem autolysis). Further information on these specimens is detailed in Ackermans and colleagues (2022) [22].

### 2.2. MRI and DTI

Image acquisition was performed using a whole-body 7T MRI scanner (Magnetom, Siemens Healthineers, Erlangen, Germany) and a 1-channel-transmit 32-channel-receive radiofrequency head coil (Nova Medical, Wilmington, MA, USA).

The fixed hemispheres, while still immersed in formalin, were exposed to a vacuum of −1 bar and agitated for 30 min. The hemispheres were then removed from formalin and placed together into a custom-built 7T ex vivo brain-imaging vessel [23]. The vessel was filled with Fluorinert (FC-770, TMC industries, Waconia, MN, USA), the vessel lid was secured with machine screws and Kapton tape, two vent ports in the lid were opened, and the container was again exposed to a vacuum of −1 bar and agitated for 10 min. Following this, residual formalin was drained off, the Fluorinert level was topped off, and the vent ports were closed and sealed with Kapton tape. The specimen container was placed into the radiofrequency coil with the brain in an orientation consistent with head-first supine orientation in a human, and inserted into the scanner. Localizer images were acquired, and iterative magnetic field shimming was performed by 3D phase mapping. 

High-resolution anatomical images were acquired with 3D phase-cycled balanced steady-state free precession (PC-bSSFP) and 3D multi-echo gradient echo (ME-GRE) with root-sum-of-squares echo recombination. The PC-bSSFP acquisition parameters were: field of view (FOV) = 196 × 156 × 128 mm, voxel resolution = 250 µm^3^ isotropic, repetition time (TR) = 8.01 ms, echo time (TE) = 4.01 ms, flip angle (FA) = 30°, bandwidth (BW) = 277 Hz/pixel, parallel imaging acceleration by generalized autocalibrating partially parallel acquisition (GRAPPA) [24] with acceleration factor R = 2, 2 acquisitions per phase increment, phase increment cycle = {0°, 180°, 90°, 270°}, scan time = 67 min, and recombination of individual phase increment images by root-sum-of-squares. ME-GRE acquisition parameters were: field of view = 192 × 156 × 134 mm, voxel resolution = 380 µm isotropic, TR = 38 ms, TE = {5, 10, 15, 20, 25, 30} ms with bipolar readouts, FA = 18°, BW = 260 Hz/pixel, GRAPPA R = 2, 1 signal average, scan time = 47 min 56 s, and recombination of individual echo images by coregistration using FSL FLIRT [25,26] followed by root-sum-of-squares.

Diffusion-weighted imaging was performed using diffusion-weighted 3D steady-state free precession (DW-SSFP) [27] with the following acquisition parameters: field of view = 204 × 179 × 150 mm, voxel resolution = 850 µm isotropic, TR = 29 ms, TE = 21 ms, FA = 24°, BW = 393 Hz/pixel, echo-planar imaging factor = 3, echo spacing = 3.09 ms, diffusion encoding gradient amplitude = 52 mT/m and duration 13.56 ms for a q-value of 300 cm^−1^ (corresponding to an estimated b-value of 3594 s/mm^2^), 120 diffusion encoding directions, six eddy-current-matched b = 0 acquisitions with diffusion encoding gradient amplitude = 52 mT/m and duration = 0.92 ms for a q-value of 20 cm^−1^, and scan time = 12 h 36 min. Eddy-current correction was performed using FSL eddy [28], and preliminary diffusion-tensor analysis was performed using FSL dtifit.

Diffusion tractography was performed using DSI Studio (http://dsi-studio.labsolver.org, accessed on 22 May 2022). A deterministic fiber-tracking algorithm [29] was used. Seeding, ending, and regions of interest (ROIs) were placed in the vestibulocochlear nerve (AN), VCN, IC central nucleus and external cortex, IC brachium, and the medial geniculate nucleus (MGN) using imported, manually segmented regions from the ITK-Snap software (www.itksnap.org), which automatically calculated their volumes [30]. Seed-to-end, seed-to-terminative and end-to-end connections were evaluated for each nucleus of the brainstem and mesencephalic auditory pathway. The anisotropy threshold was 0.1. The angular threshold was 60°. The step size was randomly selected from 0.5 voxel to 1.5 voxels. Tracks with a length shorter than 20 or longer than 300 mm were discarded. A total of 1,000,000 seeds were placed for each tract calculation.

### 2.3. Western Blot and BLAST

Brain tissue (about 30 mg) was homogenized in 0.1 M Tris HCl buffer pH 7.4, containing 0.15 M NaCl, 5 mM EDTA, 1% Triton × 100/0.1% SDS and Halt protease and phosphatase inhibitor cocktail (Pierce, Rockford, IL, USA). Protein concentration was determined with BCA reagent (Pierce). Protein samples (50 µg) were separated by SDS-PAGE and blotted onto polyvinylidene difluoride (PVDF) membranes (Millipore-Sigma, Burlington, MA, USA). Blots were blocked with 25 mM Tris HCl, pH 7.5, 0.15 M NaCl, 0.1% Tween-20 (TBST), 5% nonfat dry milk and probed overnight with the relevant primary antibody diluted in blocking solution. Primary antibodies used for Western blots were the same as those for immunohistochemistry and are listed in Table 1. Blots were then incubated for 1.5 h with the appropriate horseradish peroxidase (HRP) conjugated secondary antibody (Cytiva, Marlborough, MA, USA: anti-rabbit NA934 and anti-mouse NA 931, and ThermoFisher Scientific, Waltham, MA, USA: anti-rat A18749 antibodies, respectively) diluted in blocking solution (1:7500). The bands were visualized by ECL Prime (Cytiva, RPN 2232) and imaged with an ImageQuant 800 imaging station (Cytiva), with a Precision Plus Protein All Blue prestained protein standard ladder (Biorad 1610373) on either side of the samples. In case of inconclusive results with Western blot, the basic linear alignment search tool (BLAST) was implemented as previously reported [5] to confirm antibody specificity.

### 2.4. Immunohistochemistry

Tissue blocks comprising the entire right (Marine 0116) and left (Marine 0142) IC and VCN were sampled from the formalin-fixed brains. Only the left hemisphere was available for Marine 0142, and the caudal part (about one-third) was missing. Brain sections from Marine 0116 were discarded following the pilot studies due to tissue deformation when drying on the slide. Following several washes in phosphate-buffered saline (PBS, pH 7.0), the blocks were cut coronally into 50 µm-thick sections using a vibratome (Leica VT1000S) and stored in solution with 0.1% sodium azide. Every 30th serial section of the LIC and every 20th section of the VCN was used for testing different antibodies that had elicited an immunoreactivity in pilot immunohistochemistry runs, which included dolphin primary auditory cortex sections. Antibodies that were initially tested but then excluded due to lack of reactivity can be found in Annex 1, Table A1. The antibodies chosen for stereology included anti-Aβ (ThermoFisher Scientific, #700254, diluted 1:500), anti-fibronectin (Sigma-Aldrich, St. Louis, MO, USA: #F3648, 1:200), anti-TDP-43 (Biolegend, San Diego, CA, USA: #829901, 1:200) for neurons, antibody SMI-312 (Biolegend, #837904, 1:1000) against medium- and heavy-chain phosphorylated neurofilament proteins (pNFP) in axons, anti-Iba-1 (Wako, Neuss, Germany: #019-19741, 1:500) for microglia, and anti-GFAP (Dako, Düsseldorf, Germany: #Z0334, 1:500) for astrocytes. Appropriate secondary antibodies—biotinylated goat anti-rabbit (Vector Laboratories, Newark, CA, USA: #BA-1000-1.5) for Aβ, fibronectin, GFAP and Iba-1, biotinylated goat anti-rat (Vector Laboratories; #BA-9400-1.5) for TDP-43 and biotinylated donkey anti-mouse (Jackson ImmunoResesarch, West Grove, PA, USA: #715-065-150) for pNFP, respectively—were applied. Primary antibodies were omitted in concomitant negative control sections.

### 2.5. Microscopy and Stereology

Brightfield microscopy images and scans were taken on an Axiophot brightfield microscope (Carl Zeiss Microscopy, Jena, Germany), with a 20×/0.8 Plan-Apochromat and 40×/1.3 Oil Plan-Neofluar objective for immunohistochemical sections, and a 40×/0.6 Korr Plan-Neofluar objective for the archived Nissl sections using StereoInvestigator (version 11.03, MBF Bioscience, Williston, VT, USA).

Contours were traced around the whole of the left IC, and separate contours marked the neuron-dense, putative central IC nucleus (CN) and the surrounding tectosomes or external cortex (EC). The VCN was contoured in its entirety. The same procedure was followed for the right IC and VCN of Dolphin 2, whose Nissl-stained, serial, 35 µm-thick and celloidin-embedded coronal brain sections were assessed for differences in cell counts and densities arising from alternative tissue processing.

The volume of each subnucleus was calculated using the Cavalieri probe as recently reported by Nieder and colleagues (2022) [13], and the coefficient of error [31] calculated for each subnucleus was less than 0.09. Obtained volumes from processed tissue and from the MRI scans of the available brain hemispheres (left and right in Marine 0116; only left in Marine 0142) is represented in Table 2.

Image stacks in a systematic random sampling grid were acquired with a Z-step of 2 µm. The top of every sampling site was focused on manually. The optical fractionator workflow probe in StereoInvestigator (grid size 2000 × 2000 µm for the IC and 1000 × 1000 µm for the VCN, SRS grid layout at 100% of the region of interest, optical disector height 18 µm with 1-µm top and bottom guard zones, manual focus) was used on a series of 30 sections for IC and 20 sections for VCN, each separated by 1500 µm in the IC and 1000 µm in the VCN. For amyloid beta, TDP-43 and fibronectin, a counting frame of 150 × 150 µm was used, and neurons with nuclear, cytoplasmic, both, or no immunoreactivity were counted using different markers in StereoInvestigator. For Iba-1 and GFAP, a counting frame of 30 × 30 µm was used and cytoplasmic immunoreactivity of microglia and astrocytes, respectively, was counted. In the case of pNFP, the axonal immunoreactivity and length density of the axons was recorded using a Spaceball probe with a radius of 18 µm.

### 2.6. Data Analysis

Data analysis was performed using Microsoft Excel (from the Microsoft 365 apps package) and R version 4.2.1. Bar charts were created using GraphPad Prism 8. Heatmaps of 3-dimensional immunohistochemical marker distribution were generated following an R-script from Ackermans and colleagues (2022) [22], publicly available on GitHub (https://github.com/NLAckermans/Ackermans2022BovidTBI/blob/main/Muskox_heatmaps_220314.Rmd, accessed on 18 June 2022).

## 3. Results

### 3.1. MRI

**Figure 1:**
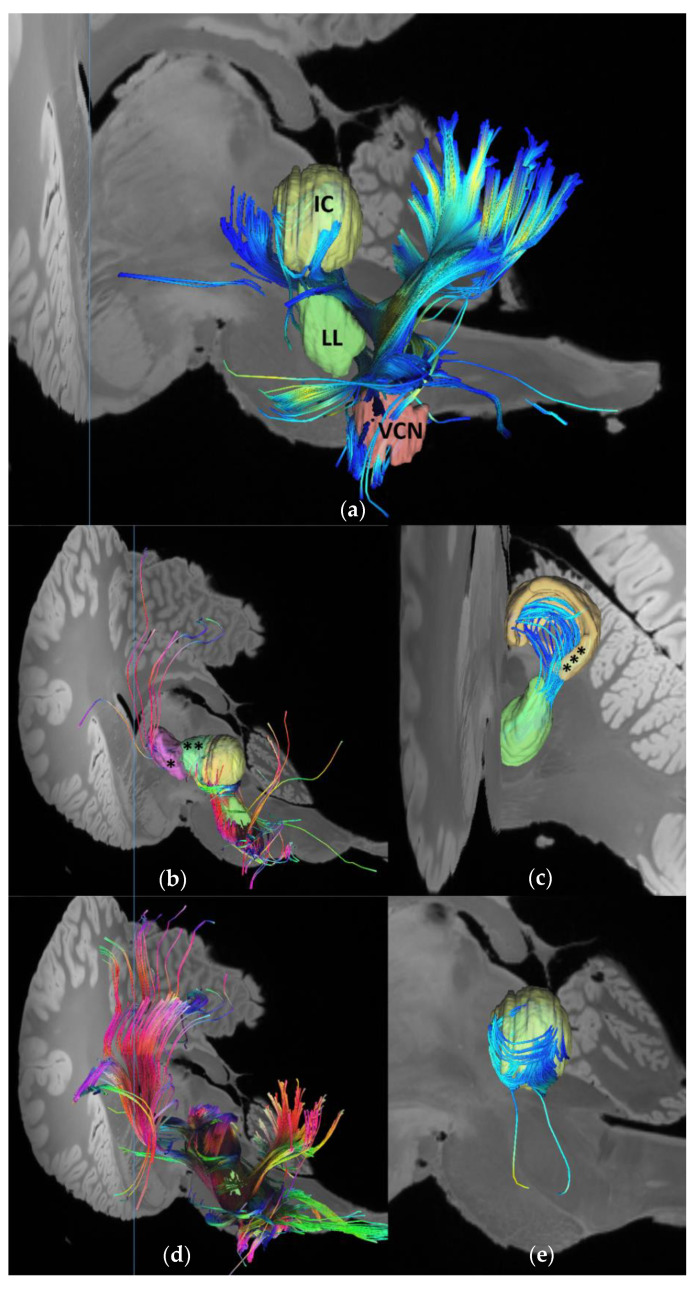
Fiber tracts between the brainstem and midbrain auditory nuclei using a deterministic tracking algorithm [29]. Number of seeds was limited to 1,000,000 in each calculation. (**a**) End-to-end connectivity between the left VCN and IC central nucleus. Total number of tracts: *n* = 63. The lateral lemniscus to accentuate the lemniscal pathway. Lateral perspective; (**b**) Fibers (*n* = 1652) calculated to end in the lateral lemniscus, with the whole brain considered as a seeding region. Caudolateral perspective. MGN (*) and IC brachium (**) are visualized for reference; (**c**) End-to-end fibers (*n* = 46) between the lateral lemniscus and the IC external cortex (***). Caudal perspective; (**d**) Fibers (*n* = 7456) calculated to pass through or end in the lateral lemniscus as a ROI, with the whole brain considered as a seeding region. A higher number of tracts is calculated in comparison with (**b**). Caudolateral perspective; (**e**) End-to-end fibers (*n* = 362) between the IC central nucleus and its external cortex (not shown to better visualize fibers). Lateral perspective.

**Figure 2:**
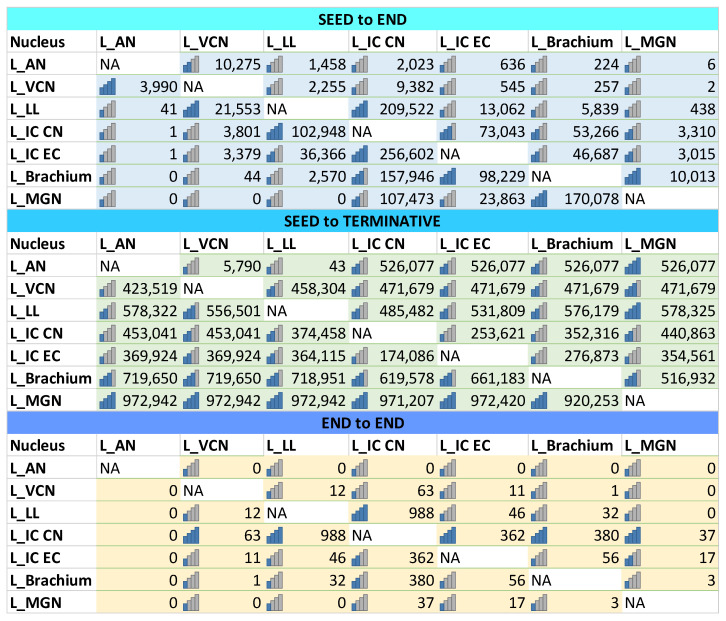
Calculated fiber tracts for seed-to-end, seed-to-terminative (ends all tracts regardless of their anatomical endings), and end-to-end connectivity of brainstem and midbrain auditory nuclei. The number of tracts is less indicative than their relative differences between the different nuclei, as their number does not correspond to the number of axons and synaptic connections.

The 7T-MRI scans enabled the identification and in situ volume estimate of unprocessed, formalin-fixed dolphin brain nuclei. Deterministic unihemispheric fiber tracking revealed strong connectivity between the auditory nuclei (VCN and lateral lemniscus and the cerebellum; Figure 1a), and the afferent auditory lemniscal pathway up to the IC, IC tectosomes, collicular brachium, and medial geniculate nucleus. The IC and its brachium were particularly rich in putative synaptic endings, corresponding to the former’s role as a central integrative hub for sensory input processing, while the latter and the medial geniculate nucleus stood out in terms of the number of passing tracts (Figure 2). Overall, the cetacean auditory pathways described in historic works were recreated on the brainstem and mesencephalic levels [32,33,34]. More fiber tracts were calculated when the nucleus in question was considered as a region of interest (ROI) rather than as an ending point (Figure 1b,d). End-to-end connections revealed the intricate wiring between the different nuclei, such as the numerous tracts between the lateral lemniscus and the IC tectosomes (Figure 1c) and within different regions of the same nucleus, such as between the IC central nucleus and its tectosomes (Figure 1d).

### 3.2. Western Blot and BLAST

**Figure A1:**
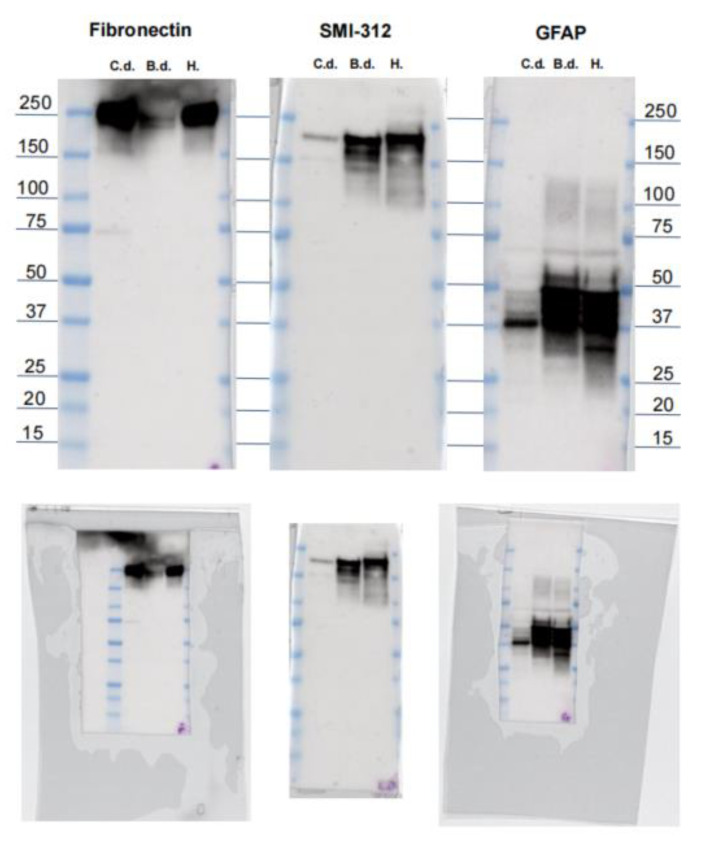
Western blot membranes from fibronectin, SMI-312, and GFAP. Top: Annotated membranes from Western blots of fibronectin, pNFP, and GFAP for C.d. = common dolphin; B.d. = bottlenose dolphin; H. = human control. Values in kDa. Bottom: original uncropped and unlabeled source images.

Western blots resulted in clear immunoreactivity against bottlenose and common dolphin and human control brains for fibronectin (~200 kDa) and GFAP (~40–50 kDa). SMI-312 revealed labeling against pNFP in all three tested species as well, showing a band at ~200 kDa and a weaker one at ~150 kDa in the human, and several intense bands between 150 and 200 kDa in bottlenose and common dolphins, corresponding to the weight of the medium and heavy neurofilament chains this antibody targets. Membrane images are provided in Appendix A, Figure A1.

TDP-43 did not show any immunoreactivity in any of the tested species with the antibodies used in this essay, and Iba-1 was not suitable for WB. A BLAST search confirmed that proteins of very similar amino acid sequence are encoded in the dolphin genome. Human TDP-43 (ABO32290.1) showed 97.83% identity with bottlenose dolphin TAR DNA-binding protein 43 (XP_019786443.1, E-Value: 0), while human Iba-1 (BAA13088.1) showed a 94.56% (E-Value: 1 × 10^−96^) homology with the dolphin allograft inflammatory factor 1 (XP_004311265.1). The Aβ antibody was tested by WB in our previous study [5] and was considered validated in bottlenose dolphins. 

### 3.3. Immunohistochemistry and Stereology

The six tested antibodies all showed clear immunoreactivity in the human control and bottlenose dolphin IC and VCN tissue. Pre-and post-processing IC and VCN volumes estimated from 7T-MRI scans and the Cavalieri probe from serial sections are reported in Table 2. Cell densities below are reported in reference to the Cavalieri volumes in Marine 0142. 

**Figure 3:**
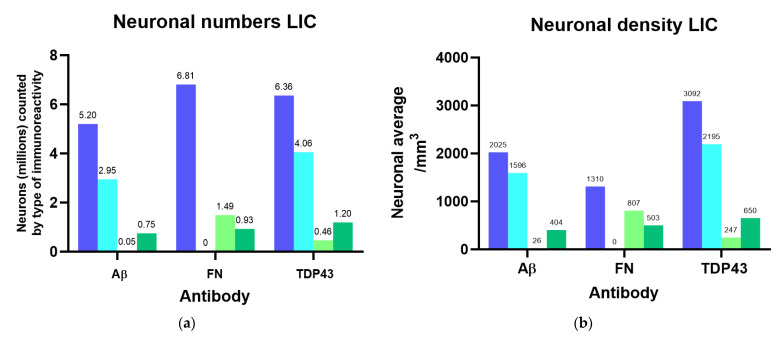

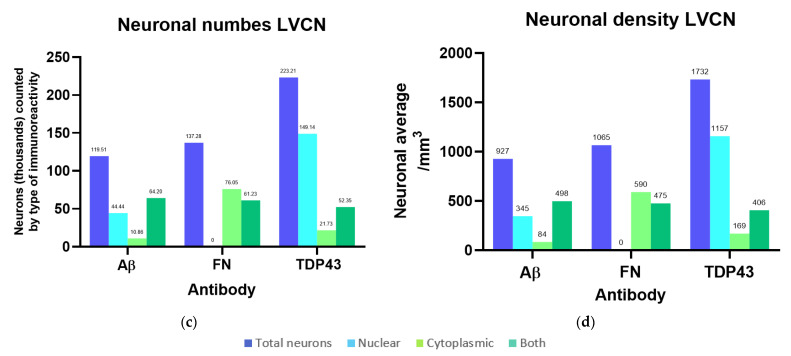
Neuronal numbers and their densities for the left IC and VCN considering different immunoreactivity patterns for Aβ, fibronectin (FN), and TDP-43. (**a**) Total number of neurons counted in each IC probe compared to nuclear, cytoplasmic, or both nuclear and cytoplasmic immunoreactivity; (**b**) Total density of neurons counted in each IC probe compared to nuclear, cytoplasmic, or both nuclear and cytoplasmic immunoreactivity referring to the IC Cavalieri volume; (**c**) Total number of neurons counted in each VCN probe compared to nuclear, cytoplasmic, or both nuclear and cytoplasmic immunoreactivity; (**d**) Total density of neurons counted in each VCN probe compared to nuclear, cytoplasmic, or both nuclear and cytoplasmic immunoreactivity referring to the VCN Cavalieri volume.

Since the optical dissector probes for Aβ, fibronectin, and TDP-43 all considered non-immunoreactive (ir) neurons and therefore yielded independent estimates of total neuronal numbers and densities for the same nuclei, their results were averaged to obtain an estimate of 6.13 million neurons with 3313 neurons/mm^3^ in the IC (4.85 million ± 754.58 thousand in the CN with a density of 3561/mm^3^; 1.28 million ± 159.71 thousand in the EC, with 2621/mm^3^), and 3.16 million ± 102.19 thousand neurons with 2455/mm^3^ in the VCN. The calculations of the percentages and densities of neurons with different types of immunoreactivity (nuclear/cytoplasmic/both nuclear and cytoplasmic) are reported in full in Supplementary Table S1, and all number estimates from StereoInvestigator probes are summarized in Table A2. Neuronal numbers and densities according to the observed types of immunoreactivity are visualized in Figure 3.

**Figure 4:**
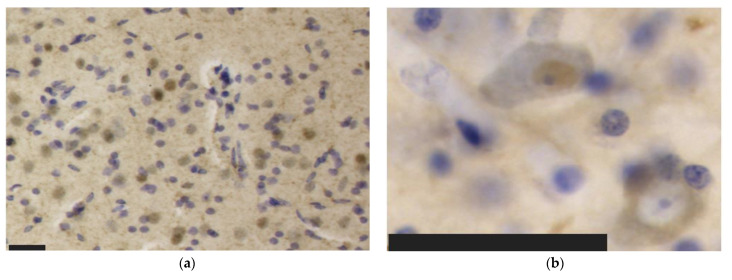

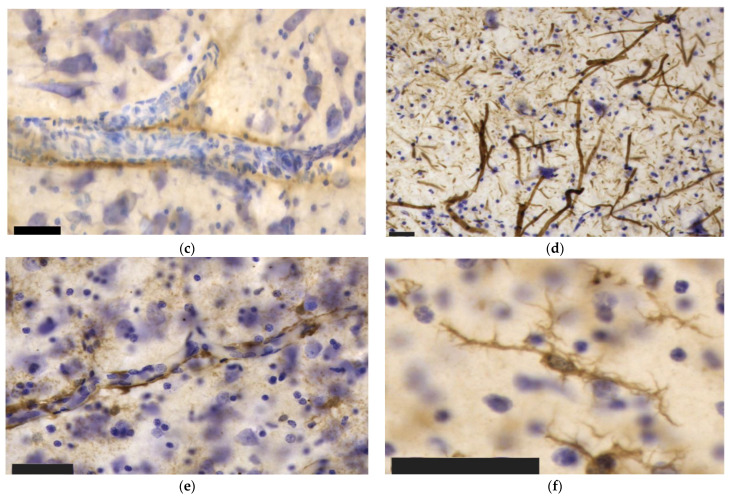
Immunohistochemical patterns of the antibodies tested in this study in the bottlenose dolphin brain. Scale bar: 50 µm. (**a**) Aβ reactivity in neuronal nuclei and in the neuropil of the IC; (**b**) Nuclear (top left) versus cytoplasmic (bottom right) neuronal immunoreactivity to TDP-43 in IC neurons; (**c**) Perivascular immunoreactivity TDP-43 in the IC; (**d**) pNFP-immunolabeled thinner axonal fibers of the IC central nucleus alternating with much thicker external cortex axons; (**e**) Perivascular and intraparenchymal GFAP-expressing astrocytes in the IC; (**f**) Though the ramified morphology prevailed, few rod-shaped Iba-1-ir microglia were present in the VCN.

The main types of immunoreactivity observed in the tested dolphin tissues are displayed in Figure 4.

#### 3.3.1. Aβ

Aβ was observed in 71% of the neuronal nuclei of the IC central nucleus (Figure 4a) and in 7% of its external cortex neurons, where most of its immunoreactivity was present simultaneously in the nucleus and cytoplasm (48%). In total, 56% of all IC neurons displayed immunoreactivity in the nucleus, 14% in both nucleus and cytoplasm, and 0.9% only in the cytoplasm. In the VCN, only 18% of neurons showed only nuclear immunoreactivity, 26% nuclear and cytoplasmic, and 4% cytoplasmic alone. Mild diffuse pericapillary immunoreactivity was too faint to be quantified. A few glial cells also appeared IR, although without confocal microscopy, it was not possible to define whether these were micro- or macroglia.

#### 3.3.2. Fibronectin

**Figure 5:**
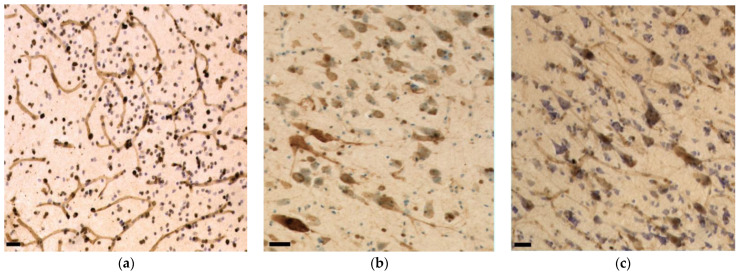
Fibronectin immunoreactivity in dolphin and human brain cortex. (**a**) Vascular and glial immunoreactivity in a healthy human brain; (**b**) Neuronal cytoplasmic and nuclear immunoreactivity in the primary auditory cortex of a healthy bottlenose dolphin brain; (**c**) Predominantly neuronal and very little vascular immunoreactivity in the blood vessels of a human brain with CTE.

Fibronectin appeared only in the cytoplasmic or nuclear and cytoplasmic compartments simultaneously in the examined dolphin. The former pattern was seen in 23%, 17%, and 18%, and the latter in 14%, 13%, and 14% of the IC central nucleus, its external cortex, and the VCN, respectively. A total of 36% of IC and 32% of VCN neurons showed fibronectin immunoreactivity. Some glial cell nuclei were also fibronectin-ir. A human cortical section (Brodmann area 11) from a control case was used as a positive control for fibronectin (Figure 5a). Fibronectin coated the capillary walls and could be observed in most glial and a few neuronal cell nuclei. Unexpectedly, a comparable immunohistochemical pattern was observed in cortical samples from the bottlenose dolphin (Figure 5b; primary auditory cortex) and a human with CTE (Figure 5; superior frontal cortex) revealing very weak capillary immunoreactivity, little (bottlenose dolphin) or no (human with CTE) glial immunoreactivity, and mostly neuronal cytoplasmic and nuclear signal.

#### 3.3.3. TDP-43

As with Aβ, TDP-43 immunoreactivity was observed in neuronal nuclei or cytoplasm alone (Figure 4b), or in both compartments simultaneously. Predominant nuclear immunoreactivity was observed in the IC central nucleus (67%), external cortex (53%), and VCN (55%), followed by simultaneous nuclear and cytoplasmic immunoreactivity at 17%, 26%, and 19% and a cytoplasmic pattern in 7%, 7%, and 8% of the cells, respectively. Few vessels displayed mild perivascular immunoreactivity (Figure 4c).

#### 3.3.4. pNFP

**Figure 6:**
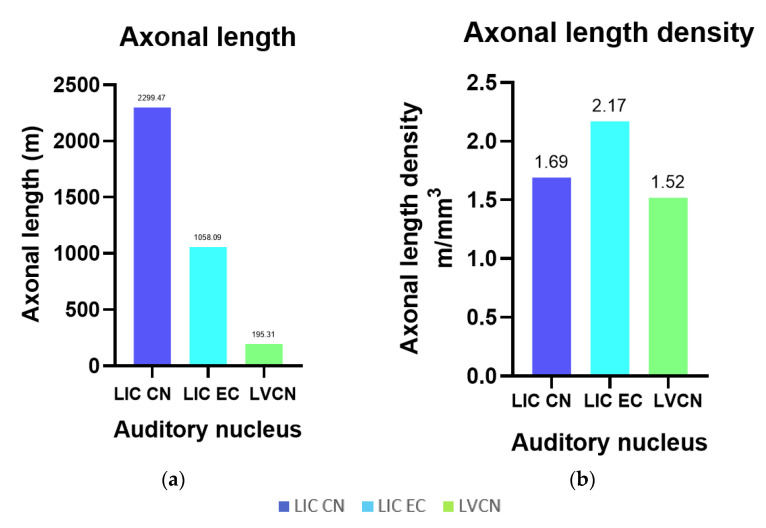
Axonal length and their densities for the left IC central nucleus (CN) and external cortex (EC) and VCN. (**a**) Total axonal length in the LIC CN and EC, and in the VCN; (**b**) Total length density of axons counted in the LIC CN and EC, and in the VCN, referring to the IC Cavalieri volume.

The axons of the VCN and IC external cortex appeared as very thick, pNFP-ir filamentous structures (Figure 4d). The IC central nucleus mostly consisted of thinner and shorter filaments. Axonal length was estimated to be 2299.47 m in the IC central nucleus, 1058.09 m in its external cortex and 195.31 m in the VCN, corresponding to the structures’ different volumes (Figure 6a). Axonal length density was, respectively, 1.69 m/mm^3^, 2.17 m/mm^3^, and 1.52 m/mm^3^ in the IC central nucleus, external cortex, and VCN (Figure 6b). The higher length density of the IC external cortex corresponds to the assumption that many afferent fibers reach the IC from here [33,35].

#### 3.3.5. Iba-1

**Figure 7:**
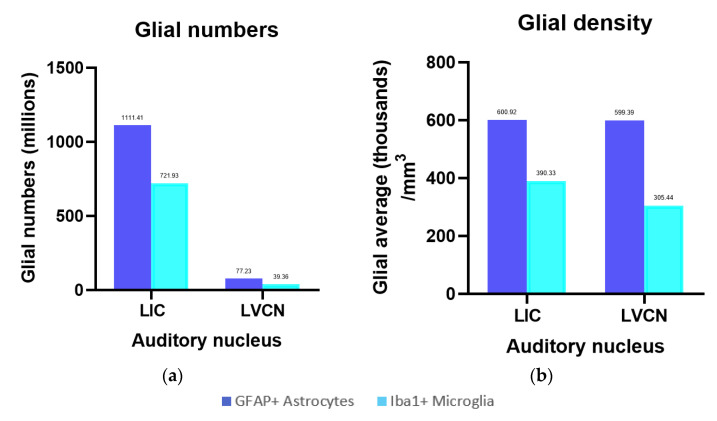
Glial numbers and their densities for the left IC and VCN. (**a**) Total number of GFAP-containing astrocytes and Iba-1-ir microglia in the left IC and VCN; (**b**) Total density of GFAP-expressing astrocytes and Iba-1-ir microglia in the left IC and VCN referring to the IC Cavalieri volume.

Iba-1 was localized in all parenchymal microglia (intravascular monocytes were not counted). The numbers counted in the IC central nucleus, external cortex and VCN totaled 405.63 million, 316.30 million, and 39.36 million, with their respective densities calculated at 297.85 thousand, 648.62 thousand, and 305.44 thousand microglia/mm^3^ (Figure 7a). On average, the total microglial density in the IC is 390.33 thousand/mm^3^. As such, it is in the same order of magnitude as that of the VCN (Figure 7b). Most of the observed microglia displayed a filamentous to dendritic morphology, suggesting that the animal did not display evident neuropathology, although few rod-shaped microglia were present in both IC and VCN (Figure 4f).

#### 3.3.6. GFAP

GFAP-expressing astrocytes made up a population of 717.04 million (IC central nucleus), 394.37 million (IC external cortex), and 77.23 million (VCN) cells (Figure 7a). Their density in the three regions amounted to 526.51 thousand, 808.72 thousand, and 599.39 thousand astrocytes/mm^3^, respectively, and as with Iba-1, the averaged IC density (600.92 thousand/mm^3^) was comparable to that of the VCN (Figure 7b).

### 3.4. Marker Distributions

**Figure 8:**
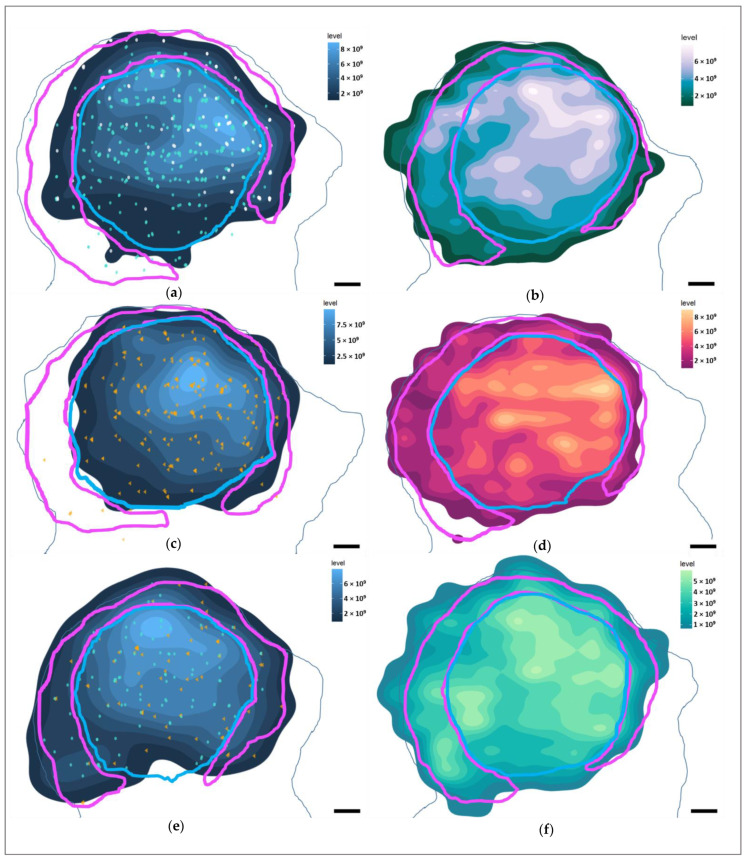
Heatmaps of marker distributions in the left IC of Marine 0142 in the coronal plane (caudal perspective). In light blue—the average contour surrounding the putative IC central nucleus area every 30th serial section, while the light pink contour represents the putative IC external cortex. Lighter colors of the heatmaps represent a higher marker density. Scale bar: 2 mm (**a**) Heatmap of all Aβ-containing neurons. Turquoise points—neurons with nuclear immunoreactivity. White points—neurons with simultaneous cytoplasmic and nuclear immunoreactivity. (**b**) Heatmap of pNFP-ir axons. (**c**) Heatmap of all fibronectin-ir neurons. Orange points—neurons with cytoplasmic immunoreactivity pattern. (**d**) Heatmap of GFAP-expressing astrocytes. (**e**) Heatmap of all TDP-43-containing neurons. Points as in (**a**,**c**). (**f**) Heatmap of Iba-1-expressing microglia.

**Figure 9:**
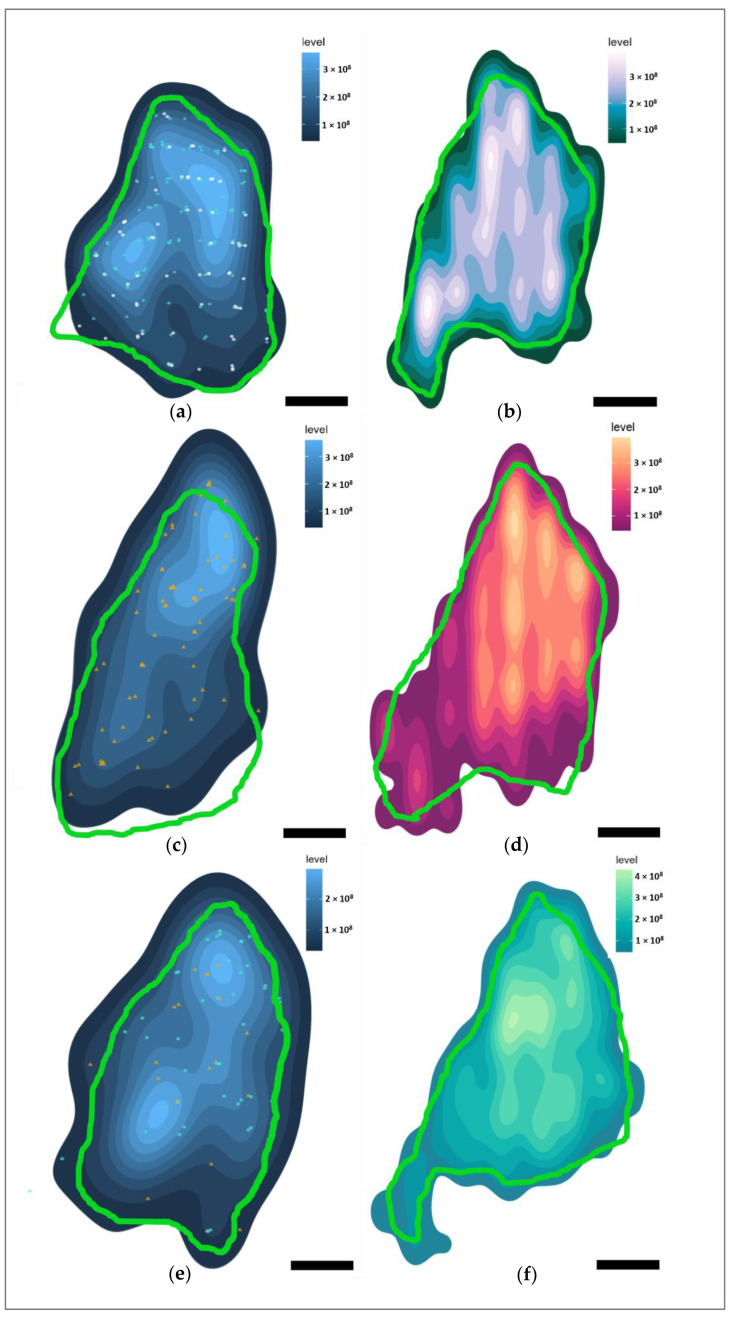
Heatmaps of immunohistochemical marker distributions in the left VCN of Marine 0142 in the coronal plane (caudal perspective). In green—the average contour surrounding the putative VCN area from every 20th serial section. Lighter colors of the heatmaps represent a higher marker density. Scale bar: 2 mm. (**a**) Heatmap display total Aβ-containing neurons, regardless of immunoreactivity type. Turquoise points—neurons with nuclear immunoreactivity. White points—neurons with simultaneous cytoplasmic and nuclear immunoreactivity. (**b**) Heatmap of pNFP-ir axons. (**c**) Heatmap of all fibronectin-ir neurons. Orange points—neurons with cytoplasmic immunoreactivity pattern. (**d**) Heatmap of GFAP-ir astrocytes. (**e**) Heatmap of all TDP-43-ir neurons. Points as in (**a**,**c**). (**f**) Heatmap of Iba-1-ir microglia.

With the use of stereological data, it was possible to create heatmaps from 3-dimensional data to visualize the distribution of the immunolabeled neurons, axons, and glia in Marine 0142 (Figure 8 for the IC, Figure 9 for the VCN).

Most of the IC neurons were concentrated in the dorsomedial segment of the IC central nucleus, and this was reflected in the distribution of the neuron-associated markers (Figure 8a,c,e). In the VCN, this distribution was somewhat more homogeneous (Figure 9a,e) for Aβ and TDP-43, and reflected the glove-like morphology of the VCN surrounding the incoming cochlear nerve fibers. Fibronectin appeared to be concentrated in the dorsomedial VCN segment (Figure 9c).

Regarding the different immunoreactivity patterns, intranuclear Aβ was mostly restricted to the IC central nucleus, and less abundant in the external cortex and the VCN. Fibronectin immunoreactivity was observed mostly in the IC central nucleus, particularly with regards to its cytoplasmic expression. VCN distribution was homogenous. 

TDP-43, on the other hand, was equally distributed between the central nucleus and external cortex of the IC and the VCN, with comparable nuclear and cytoplasmic immunoreactivity between the three subnuclei (58% nuclear; 21% nuclear and cytoplasmic; 7% cytoplasmic out of all the counted neurons).

pNFP appeared concentrated in the dorsomedial and ventrolateral regions of the IC central nucleus (Figure 8b), but also in the dorsal and lateral segments of the tectosomes, likely reflecting the main afferent and efferent fibers of the IC, which corresponds to the fiber tracks observed in the DTI data (Figure 1d,e). In the VCN, pNFP-ir fibers appeared interspersed with the neuronal somata, although it must be kept in mind that this nucleus was not complete in the examined specimen. 

Glial distributions (Figure 8d,f and Figure 9d,f) lacked large concentration gradients, being somewhat denser in the dorsomedial segments of both auditory nuclei. 

### 3.5. Intra- and Interspecies Comparisons

**Figure 10:**
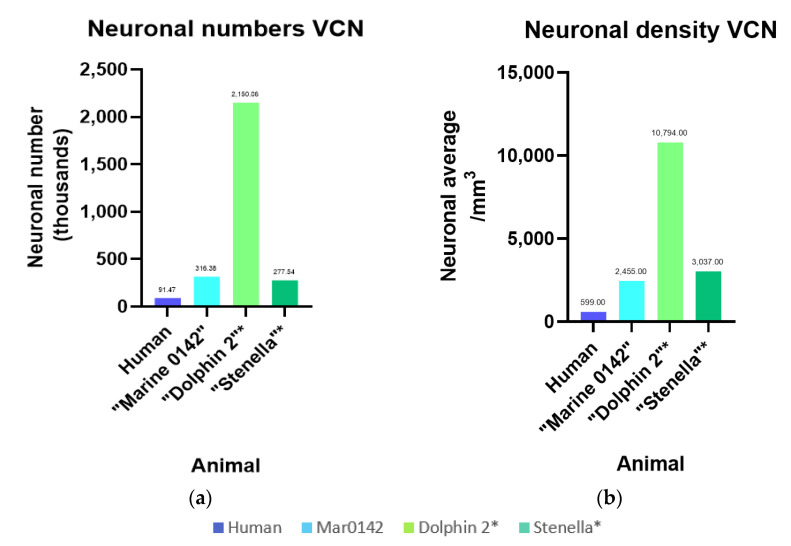
Intra- and interspecies comparison of numbers and densities for the VCN. Human values were taken from existing literature [37]. * Bottlenose Dolphin 2 and the striped dolphin (*Stenella*) VCN neurons were counted using archived, Nissl-stained, celloidin-embedded sections. (**a**) Total number of neurons counted in the VCN; (**b**) Total density neurons referring to the VCN Cavalieri volume.

To our knowledge, this is the first stereological estimates of neurons and glia ir against the investigated antibodies in the bottlenose dolphin IC and VCN. While the two hemispheres of Marine 0116’s brain yielded high-quality 7T-MRI scans, the tissue quality made it unsuitable for immunohistochemistry. The celloidin-embedded Nissl-sections of the right IC and VCN of Dolphin 2 (a bottlenose dolphin; [36]) and the right VCN of a striped dolphin (*Stenella coeruleoalba*) were analyzed for comparison. Values for cochlear nucleus numbers (subdivisions in a dorsal and VCN are indistinct) have previously been reported for healthy individuals versus people with presbycusis by Hinojosa and colleagues (2011) [37], and the former number and density is presented in Figure 10 in comparison with the cetaceans. At 91.47 thousand neurons, human values are about a third (31%) of Marine 0142’s VCN (316.38 thousand). The difference is exorbitantly greater when comparing Marine 0142, which was processed minimally and embedded using DPX, to Dolphin 2 (2.15 million neurons), whose brain volume presumably shrunk by at least 50% due to celloidin embedding, thus reflecting on the neurons observed per counting frame and density estimates. While the striped dolphin’s brain was processed similarly to Dolphin 2’s, its numbers (personal communication, Ava Akbarian, Hof laboratory) are similar to those of Marine 0142, despite its physiologically, species-characteristic smaller brain volume [38].

IC neuronal numbers were only available for Marine 0142 and Dolphin 2 (8.30 million—IC central nucleus, 1.76 million—external cortex, 2.15 million—VCN), and revealed 2-7 times higher values in the celloidin-embedded bottlenose dolphin tissue.

## 4. Discussion

This study reports a multimodal and quantitative approach to assessing cetacean auditory nuclei, expanding the knowledge of neurochemical signatures and brain connectivity. Stereological probes for systematic random sampling yielded reliable cell counts with coefficients of error (CEs) < 10% for most of the tested markers with regards to total immunoreactivity.

Following our previous work, markers of interest were chosen according to their relevance in the neurodegenerative and neuroprotective processes, as well as in plasticity. Aβ was consistently observed in the nuclei of IC and VCN neurons in 21 bottlenose dolphins (including the specimens from Orekhova and colleagues, 2022 [5]), and the present work shows that intranuclear Aβ is present in 71% of IC central nuclear cells, pointing to a widespread, potentially physiological pattern in a healthy dolphin and matching findings in other cetacean species [14]. 

As previously described by these authors, a potential neuroprotective role of nuclear Aβ oligomer may facilitate nuclear degradation of misfolded proteins [39,40], particularly assuming repetitive exposure to mild hypoxia that may be experienced by animals with dive intervals of up to ~8 min [41,42]. This hypothesis is worthy of further investigation, as extracellular Aβ aggregation is correlated with AD and used for staging of the disease [43], yet no clear causality has been described [44]. 

In this context, the localization of fibronectin is of particular interest. Contrary to the expected capillary reactivity, as it was seen in the healthy human control tissue, the bottlenose dolphins examined for this study displayed neuronal cytoplasmic (with and without nuclear signal) and limited glial immunoreactivity in the IC, VCN, and auditory cortex. This corresponded to the pattern seen in a human CTE brain. 

As a component soluble in the extracellular matrix (ECM) and insoluble when built into vascular basal membranes, fibronectin is secreted by endothelial, pericytic, and astroglial cells [16]. It may also be expressed on the neuronal cell surface along with other adhesive glycoproteins as part of the ECM, enabling integrin-associated signal transduction and thus reflecting the activity of neuronal, glial, and vascular cells that are responsible for its proteolytic degradation [45]. Following neuronal insults such as stroke and traumatic brain injury (TBI), the hybrid ECM proteins are shown to be acutely down- and chronically upregulated within the scar tissue, shifting from the perivascular to the perineuronal compartment and putatively enabling plastic adaptations to take place. As such, perineuronal immunoreactivity is expected in a CTE brain.

Wang and colleagues (2013) attribute a key neuroprotective role to the “fibronectin-integrin-growth factor receptor-signal transduction-gene and protein expression cascade”, highlighting its capacity to compensate TBI-induced synaptic deficits by modulating neuron-glial extrasynaptic transmission [20]. Furthermore, ECM homeostasis is thought to be disturbed as a consequence of hypoxia in fibrotic breast, mesenchymal stem cells, and other tissues [46,47] as well as ischemic conditions, although in the latter case, immunoreactivity is largely restricted to perivascular and diffuse parenchymal and not cell-associated immunoreactivity pattern [48,49]. Moreover, hypoxia may also be a driver of neuroinflammation by promoting Aβ build-up and disrupting calcium homeostasis when the tissue is exposed chronically, although intermittent and acute exposure tends to induce neuroprotective mechanisms [50].

Considering this together with higher myelination [9] and antioxidant protein levels [10] in deeper- and thus longer-diving cetacean species, and generally higher neuroglobin levels in cetaceans versus terrestrial mammals and seals [51], perineural fibronectin appearance in a healthy dolphin brain concurrently with intranuclear Aβ is remarkable and suggests that a neurochemical signature reminiscent of hypoxia may be physiological in dolphins. However, a reliable vascular marker would still be important despite the lack of immunoreactivity in the antibodies tried in this study (Appendix A, Table A1), as TBI may cause chronic vessel-associated neuroinflammation [52].

TDP-43 appeared on average in 87% of counted auditory nuclei neurons, mostly in nuclear (58%), followed by nuclear and cytoplasmic (7%), and only cytoplasmic (21%) patterns. In a recent study on the brains of seven free-ranging common dolphins variably exposed to β-*N*-methylamino-L-alanine (BMAA) and methylmercury, all the specimens were observed to have neuronal cytoplasmic inclusions morphologically reminiscent of AD disease in humans, widespread in different cortical areas and layers independent of BMAA exposure levels, but potentially associated with a synergistic pathology in combination with high methylmercury levels [4]. Furthermore, *TARDBP*, the gene encoding for TDP-43, was found to be upregulated in these dolphins [4]. However, no quantitative assessment was available for the protein-expression data. 

In humans with Guamanian Parkinsonism-dementia complex, hippocampal CA1 neuronal loss was correlated with significantly lower numbers of neurons expressing nuclear TDP-43 and higher numbers of cytoplasmic TDP-43. Healthy controls displayed 43.7% of positive nuclei compared to 22% in this diseased cohort [53]. While human values for the IC and cochlear nuclei are unavailable, the intranuclear 58% in Marine 0142 auditory nuclei may reflect the lack of neuropathological changes in this dolphin, as confirmed by microscopic examination. 

Qualitative descriptions of TDP-43 neuronal mislocalization from the nucleus to the cytoplasm in various neurodegenerative disorders abound [54,55], and thus it may be a protein with a similar apoptosis-heralding dynamic, but potentially more chronic expression in the dolphin than the previously described diacylglycerol-ζ [5].

While antibodies against cytoskeleteal neurofilament have successfully been used in cetaceans (SMI-32—[13,56]; NF200—[5,57]), they mostly visualize somatic and dendritic elements. Here we used pNFP to analyze axonal length and density, targeting heavily phosphorylated axonal epitopes [58], which may thus serve as a reference for comparison with other individuals and species in an evolutionary and pathophysiological context. 

Validating pNFP for cetaceans may help assess oxidative axonopathy by assessing the ratio of phosphorylated neurofilaments to total neurofilament proteins (using NF200). This has been shown in an acute loss of phosphorylation, quantifying the loss of axonal function in acute oxidative injury [59] and correlating with the duration of some neurodegenerative diseases such as multiple sclerosis [60]. 

GFAP-expressing astrocyte and Iba-1-expressing microglial numbers and densities may also serve as a baseline, as both cell types are involved in the response to pathogen- [61,62], toxin- [63], age-related, and immune-mediated neuroinflammation [64], and have even been involved in acoustic trauma [65,66,67]. Future studies may focus on quantifying proteins such as Aβ and fibronectin in glial cell populations using confocal/multiplexing methods, as their involvement has been verified in neurodegeneration and neuroprotection [20,68,69,70]. 

Cetaceans display remarkable cognitive abilities, including complex social relationships, mirror self-recognition, the ability to pass along cultural behaviors such as specialized hunting techniques, and even the use of different dialects, traditions, and tools [71,72,73,74]. In this regard, comparison of the reported protein expression to individuals with known cognitive decline or deafness, especially in dolphins from aquaria where behavioral assessments are available, would be very valuable venue for upcoming research. Ethically sound functional studies in live, trained dolphins, such as using fMRI, are very difficult due to a variety of logistical and training challenges. Nevertheless, they are likely to become feasible with time, providing unique insight and validation for post-mortem assessments. 

While stereological estimates of the above markers are a useful baseline for future comparisons with other individuals and species, cell numbers, and especially densities, depend heavily on the way the tissue was fixed, processed, and probed. While MRI scans do not allow a precise border delineation using cell morphology, they can be helpful to assess in situ volumes as a reference for stereological assessment. In the case of Marine 0142, relatively little processing was performed as opposed to paraffin-embedded tissue. Nevertheless, Cavalieri volume estimates were 77% (IC) and 30% (VCN) of their in situ correlates from the 7T-MRI scans. The difference in the VCN is likely owing to macroscopic damage of the caudal part of the nucleus, which prevented us from obtaining serial sections from its caudal third. For Marine 0116, whose IC and VCN were complete in both scans and sections, Cavalieri volume estimates amounted to 84% and 75% of the MRI-based volume estimates, implying that the volumes inferred from processed brain sections are systematically underestimated.

The comparison to Dolphin 2, a celloidin-embedded, archived bottlenose dolphin specimen, showed that that a processing tissue shrinkage of about 50% can create an artificial concentration of neuronal numbers of 288%, which increases the average neuronal density by 498%. Therefore, species comparisons must also be undertaken with care. Historic studies have reported quantitative cochlear nucleus volumes and neuronal numbers [75,76,77] in harbor porpoise (*Phocoena phocoena*), fin whale (*Balaenoptera physalus*), beluga whale (*Delphinapterus leucas*), and common dolphin, revealing that volumes ranged from 6–30 times that of human, and the neurons numbered 6–17 times the average healthy human value. However, many details including age, gender, and history and tissue preparation behind the whale and human specimens are variable or unknown. 

In our case, VCN Cavalieri volumes were observed to be 8.4–18.5 times that of the most recent human study [37], 12.9–28.5 times comparing to the volumes of human cochlear nuclei in Hall’s (1967) [75] study, and 23–52 times those from Gandolfi and colleague’s VCN averages (1980) [78]. Neuronal numbers were between 4.5–70.6 times those of the adult human VCN (3.3–23.5 times that of both cochlear nuclei) compared to the above studies. 

Preliminary values for the striped dolphin (celloidin-embedded) are best compared to bottlenose Dolphin 2 due to similarities in processing, and comprise 46% of its volume, 13% of neuronal numbers, and 28% of the total packing density, which may reflect the fact that its brain mass (880.01 g on average) is around 57% that of the bottlenose dolphin (1549.9 g; [79]). This variability highlights that, while quantitative estimates of morphological and neurochemical properties are desirable, many more specimens need to be examined in future using the most tissue-sparing techniques possible, and that a multimodal approach such as used here may be of great use to offset potential artifacts. 

Not as much literature is available for the comparison between IC volumes and numbers. The intraspecies trend of a concentration in both neuronal numbers and densities is repeated between Marine 0142 and Dolphin 2 as a likely consequence of celloidin-embedding, and human volumes appear to be between 3.5 to 28 times lower than in the bottlenose dolphins [80]. We could not differentiate a dorsal from an external IC cortex in our specimens; thus, comparisons to these structures were not possible [81]. 

Furthermore, 7T-MRI-based volumes confirmed that tissue processing caused a shrinkage of 15–25% in these specimens and, as such, the DTI results reported in this study must be seen as preliminary. Connectivity from the VCN and IC of the two bottlenose dolphin hemispheres largely recreated the known delphinid auditory pathway between the brainstem and midbrain nuclei [32,34,38,77,82]. Regarding the high quality of the scans and notwithstanding the limitations of evaluating single hemispheres separately, these specimens merit further detailed connectivity studies that are beyond the scope of the present paper.

As is often the case in marine mammal science, very low sample sizes limit the significance of the acquired data, and scant information on the animals’ history, age, sex, and circumstances of death limit health assessments and the interpretation of histopathological data. Cetacean sample acquisition is opportunistic due to ethical and logistic constraints, and is inherently affected by high variability in fixation times and methods, sample integrity, and processing (see Ijsseldijk and colleagues, 2019 [83] for the current protocols of general post-mortem procedures and Orekhova and colleagues, 2022 [5] for VCN, IC, and vestibulocochlear-nerve sampling protocols). Further studies should aim to process tissues in the most sparing way available to enable future comparisons to the quantitative assessments provided in this and other recent studies [4,13].

## 5. Conclusions

The quantitative, multimodal assessment of the IC and VCN of this study expand the available knowledge on cetacean auditory nuclei neurochemical signature and connectivity, facilitating the recognition of artifacts. The stereological estimates obtained herein heighten the translational potential of dolphins to model the pathophysiology of the human brain, although different responses to neurodegenerative disease may be possible in species evidently adapted to a high-pressure, low-temperature underwater environment. They may also serve as a baseline for stranded cetaceans where pathological findings are inconclusive and acoustic trauma is suspected [84], as a support to the examination of the fragile inner ear [5,85]. Cumulative and acute toxicity from environmental and anthropogenic sources may also be better quantified [4]. 

Further studies using similar validatory and multimodal approaches are necessary to solidify the results, allow for functional intra- and interspecies comparisons between other marine and terrestrial animals and create a deeper understanding of cetacean neuroanatomy, physiology, and pathology.

## Figures and Tables

**Table 1:** Antibodies validated for bottlenose dolphin brains using immunohistochemistry (IHC) and Western blotting (WB) in this study.

Antibody	Host	Reactivity	Clonality	RRID	Catalogue Number	Dilution IHC	Dilution WB
Aβ	rabbit	rat, human, mouse, bottlenose dolphin	monoclonal	AB_2532306	700254	1:500	1:500 ^1^
fibronectin	rabbit	human	polyclonal	AB_476976	F3648	1:200	1:1000
TDP-43	rat	human, Rat	monoclonal	AB_2750118	829901	1:200	1:1000
SMI-312	mouse	human, mouse, rat	monoclonal	AB_2566782	837904	1:1000	1:1000
Iba-1	rabbit	human, mouse, rat, other	polyclonal	AB_839504	019-1974	1:500	NA
GFAP	rabbit	cat, cow, dog, human, mouse, rat, sheep	polyclonal	AB_10013382	Z0334	1:500	1:1000

^1^ Aβ-concentration in WB is derived from published work [5].

**Table 2:** Comparison of IC and VCN volumes available from three bottlenose dolphin specimens.

Specimen	Auditory Nucleus	Volume MRI (mm^3^)	Volume Cavalieri (mm^3^)
Marine 0142	LIC	1775	1362
	LVCN	435	129 *
Marine 0116	LIC	1345	-
	LVCN	387	-
	RIC	1451	921
	RVCN	378	285
Dolphin 2 **	RIC	-	571
	LVCN	-	199

* This volume corresponds to the intact rostral two-thirds of the VCN. ** Dolphin 2 processed and embedded in a different way from Marine 0142 and 0116. L and R indicate the nuclei from the left and right hemisphere, respectively.

## Data Availability

Data presented in this article are available either in the article or the Supplementary Materials.

## Supplementary Materials

The following supporting information can be downloaded at: https://www.mdpi.com/article/10.3390/vetsci9120692/s1. Table S1: Excel sheet of all calculations of percentages and densities of immunohistochemical markers supplementary to this study.

## Appendix A

**Table A1:** Antibodies tried in bottlenose dolphin brains using immunohistochemistry (IHC) in the pilot study.

Antibody ^1^	Host	Reactivity	Clonality	RRID	Catalogue Number	Lowest Dilution IHC
AT-8	Mouse	Human, Mouse, Rat (etc.)	Monoclonal	AB_223647	MN1020	1:1000
CD-31	Rabbit	Human	Polyclonal	AB_726362	ab28364	1:100
Collagen IV	Rabbit	Mouse, Rat, Hamster, Cow, Dog, Human, Pig, Zebrafish, African green monkey, Chinese hamster, Syrian hamster	Polyclonal	AB_445160	ab6586	1:300
Collagen IV	Rabbit	Mouse	Polyclonal	AB_445160	ab19808	1:300
Collagen IV	Rabbit	Human	Monoclonal	AB_2801511	ab214417	1:500
CP-13	Mouse	Human	Monoclonal	AB_2314223	Davies Lab	1:1000
dMBP	Rabbit	Human, Rat	Polyclonal	AB_2140351	ab5864	1:500
GFAP	Rabbit	Mouse, Rat, Human	Monoclonal	AB_880202	ab68426	1:1000
Isolectin B4 ^2^	*Bandeiraea simplicifolia*	Non-immune origin	Unspecific binding of glycoproteins	NA	L2140	1:40
MAP-2	Rabbit	Human	Polyclonal	AB_1853945	HPA008273	1:200
MMP-9	Mouse	Human, Mouse, Rat	Monoclonal	Not available	NBP2-80855	1:300
MOAB-2	Mouse	Human	Monoclonal	AB_2895168	MABN254	1:200
NeuN	Mouse	Avian, Chicken, Ferret, Human, Mouse, Pig, Rat, Salamander	Monoclonal	AB_2298772	MAB377	1:200
PHF1	Mouse	Human	Monoclonal	AB_2315150	Davies Lab	1:500
SMI-32	Mouse	Human, Mouse, Rat	Monoclonal	AB_2715852	801701	1:1000
Vimentin	Mouse	Human, Rat	Monoclonal	AB_306239	ab8069	1:200

^1^ Antibodies yielding inconsistent or no evident immunoreactivity were tested in morphologically healthy dolphin brain tissues. Potential immunoreactivity in pathological specimens were not excluded, but these antibodies require validation in cetaceans. ^2^ Isolectin B4 is not an antibody, but only a protein that can agglutinate cells/glycoproteins/some complex carbohydrates. It agglutinated to vascular glycoproteins in the human and dolphin sections, but the signal was too weak for further use.

**Table A2:** Full table of all the coefficients or error for the stereological markers counted in this study.

Data File	Marker	Total Markers Counted	Number of Sampling Sites	Estimated Population Using Mean Section Thickness with Counts	CE (Gundersen), m = 0	CE (Gundersen), m = 1	1st Estimated CE (Schmitz- Hof)	2nd Estimated CE (Schmitz- Hof)
**Amyloid-β**								
LIC CN	Non-ir	162	239	960,000.06	0.11	0.08	0.099	0.079
LIC CN	Nuclear	484	239	2,868,148.5	0.06	0.05	0.057	0.045
LIC CN	Cytoplasmic	7	239	41481.48	0.38	0.38	0.470	0.378
LIC CN	Nuclear + cytoplasmic	33	239	195,555.56	0.20	0.18	0.202	0.174
LIC CN	All markers	686	239	4,065,185.25	0.05	0.04	0.046	0.038
LIC EC	Non-ir	14	153	82,962.96	0.28	0.27	0.370	0.267
LIC EC	Nuclear	82	153	485,925.94	0.16	0.11	0.172	0.110
LIC EC	Cytoplasmic	1	153	5925.93	1.00	1.00	1.000	1.000
LIC EC	Nuclear + cytoplasmic	95	153	562,963	0.12	0.10	0.157	0.103
LIC EC	All markers	192	153	1,137,777.88	0.1	0.07	0.109	0.072
LVCN	Non-ir	45	137	44,444.45	0.21	0.15	0.312	0.149
LVCN	Nuclear	128	137	126,419.76	0.13	0.09	0.105	0.088
LVCN	Cytoplasmic	11	137	10,864.20	0.34	0.30	0.343	0.302
LVCN	Nuclear + cytoplasmic	65	137	64,197.54	0.14	0.12	0.129	0.124
LVCN	All markers	249	137	245,925.94	0.1	0.07	0.083	0.063
**Fibronectin**								
LIC CN	Non-ir	594	235	3,520,000.25	0.05	0.04	0.049	0.041
LIC CN	Cytoplasmic	217	235	1,285,926.13	0.09	0.07	0.085	0.068
LIC CN	Nuclear + cytoplasmic	129	235	764,444.5	0.12	0.09	0.100	0.088
LIC CN	All markers	940	235	5,570,370.5	0.04	0.03	0.045	0.033
LIC EC	Non-ir	147	100	871,111.19	0.10	0.08	0.123	0.082
LIC EC	Cytoplasmic	35	100	207,407.41	0.19	0.17	0.192	0.169
LIC EC	Nuclear + cytoplasmic	28	100	165,925.92	0.22	0.19	0.222	0.189
LIC EC	All markers	210	100	1,244,444.5	0.09	0.07	0.103	0.069
LVCN	Non-ir	300	145	296,296.31	0.12	0.06	0.099	0.058
LVCN	Cytoplasmic	77	145	76,049.38	0.12	0.11	0.133	0.114
LVCN	Nuclear + cytoplasmic	62	145	61,234.57	0.14	0.13	0.137	0.127
LVCN	All markers	439	145	433,580.28	0.1	0.05	0.072	0.048
**TDP-43**								
LIC CN	Non-ir	555	248	3,288,889	0.07	0.04	0.051	0.042
LIC CN	Nuclear	74	248	438,518.59	0.12	0.12	0.118	0.116
LIC CN	Cytoplasmic	60	248	355,555.56	0.16	0.13	0.170	0.129
LIC CN	Nuclear + cytoplasmic	140	248	829,629.63	0.12	0.09	0.108	0.085
LIC CN	All markers	829	248	49,12593	0.05	0.04	0.044	0.035
LIC EC	Non-ir	130	106	770,370.5	0.14	0.09	0.150	0.088
LIC EC	Nuclear	35	106	207,407.41	0.19	0.17	0.218	0.169
LIC EC	Cytoplasmic	17	106	100,740.75	0.24	0.24	0.253	0.243
LIC EC	Nuclear + cytoplasmic	63	106	373,333.34	0.16	0.13	0.161	0.126
LIC EC	All markers	245	106	1,451,852	0.12	0.07	0.11	0.064
LVCN	Non-ir	151	140	149,135.81	0.14	0.09	0.112	0.081
LVCN	Nuclear	47	140	46,419.76	0.19	0.15	0.150	0.146
LVCN	Cytoplasmic	22	140	21,728.40	0.22	0.21	0.217	0.213
LVCN	Nuclear + cytoplasmic	53	140	52,345.68	0.29	0.15	0.148	0.137
**pNFP**								
LIC CN	Axonal	3970	213	2,299,468,544	0.06	0.02	-	-
LIC EC	Axonal	1795	76	1,058,085,632	0.09	0.03	-	-
LVCN	Axonal	1988	115	195,308,656	0.13	0.04	-	-
**Iba-1**								
LIC CN	Microglial	2738	227	405,629,632	0.06	0.02	0.035	0.019
LIC EC	Microglial	2135	122	316,296,320	0.08	0.03	0.047	0.022
LVCN	Microglial	1594	139	39,358,024	0.09	0.03	0.055	0.025
**GFAP**								
LIC CN	Astrocytic	4840	229	717,036,992	0.05	0.02	0.03	0.014
LIC EC	Astrocytic	2662	96	394,370,400	0.08	0.03	0.058	0.019
LVCN	Astrocytic	3128	137	77,234,568	0.08	0.02	0.043	0.018
